# Single-Reaction, Multiplex, Real-Time RT-PCR for the Detection, Quantitation, and Serotyping of Dengue Viruses

**DOI:** 10.1371/journal.pntd.0002116

**Published:** 2013-04-18

**Authors:** Jesse J. Waggoner, Janaki Abeynayake, Malaya K. Sahoo, Lionel Gresh, Yolanda Tellez, Karla Gonzalez, Gabriela Ballesteros, Anna M. Pierro, Paolo Gaibani, Frances P. Guo, Vittorio Sambri, Angel Balmaseda, Kumudu Karunaratne, Eva Harris, Benjamin A. Pinsky

**Affiliations:** 1 Department of Medicine, Division of Infectious Diseases and Geographic Medicine, Stanford University School of Medicine, Stanford, California, United States of America; 2 Department of Pathology, Stanford University School of Medicine, Stanford, California, United States of America; 3 Sustainable Sciences Institute, Managua, Nicaragua; 4 National Virology Laboratory, Centro Nacional de Diagnóstico y Referencia, Ministry of Health, Managua, Nicaragua; 5 Clinical Microbiology Unit, Regional Reference Centre for Microbiological Emergencies – CRREM, St. Orsola-Malpighi University Hospital, Bologna, Italy; 6 Department of Medical Microbiology, Lady Ridgeway Hospital, Colombo, Sri Lanka; 7 Division of Infectious Diseases and Vaccinology, School of Public Health, University of California, Berkeley, California, United States of America; University of California, Davis, United States of America

## Abstract

**Background:**

Dengue fever results from infection with one or more of four different serotypes of dengue virus (DENV). Despite the widespread nature of this infection, available molecular diagnostics have significant limitations. The aim of this study was to develop a multiplex, real-time, reverse transcriptase-PCR (rRT-PCR) for the detection, quantitation, and serotyping of dengue viruses in a single reaction.

**Methodology/Principal Findings:**

An rRT-PCR assay targeting the 5′ untranslated region and capsid gene of the DENV genome was designed using molecular beacons to provide serotype specificity. Using reference DENV strains, the assay was linear from 7.0 to 1.0 log_10_ cDNA equivalents/µL for each serotype. The lower limit of detection using genomic RNA was 0.3, 13.8, 0.8, and 12.4 cDNA equivalents/µL for serotypes 1–4, respectively, which was 6- to 275-fold more analytically sensitive than a widely used hemi-nested RT-PCR. Using samples from Nicaragua collected within the first five days of illness, the multiplex rRT-PCR was positive in 100% (69/69) of specimens that were positive by the hemi-nested assay, with full serotype agreement. Furthermore, the multiplex rRT-PCR detected DENV RNA in 97.2% (35/36) of specimens from Sri Lanka positive for anti-DENV IgM antibodies compared to just 44.4% (16/36) by the hemi-nested RT-PCR. No amplification was observed in 80 clinical samples sent for routine quantitative hepatitis C virus testing or when genomic RNA from other flaviviruses was tested.

**Conclusions/Significance:**

This single-reaction, quantitative, multiplex rRT-PCR for DENV serotyping demonstrates superior analytical and clinical performance, as well as simpler workflow compared to the hemi-nested RT-PCR reference. In particular, this multiplex rRT-PCR detects viral RNA and provides serotype information in specimens collected more than five days after fever onset and from patients who had already developed anti-DENV IgM antibodies. The implementation of this assay in dengue-endemic areas has the potential to improve both dengue diagnosis and epidemiologic surveillance.

## Author Summary

Dengue, or break-bone fever, is the most common mosquito-borne viral disease of humans with over half the world's population at risk for infection. Dengue has a wide spectrum of clinical manifestations, from self-limited febrile illness to fatal hypovolemic shock, and because of this, dengue is difficult to distinguish from many other infections based on clinical characteristics alone. Diagnostic testing is therefore critical to accurately identify dengue virus (DENV)-infected patients and also rule out dengue in patients with undifferentiated fever. Unfortunately, current diagnostics for early DENV detection consist of point-of-care or laboratory-based antigen tests that lack sensitivity or molecular assays that are laborious to perform or lack the test characteristics necessary for routine use. To address these limitations, we developed a single-reaction, multiplex, real-time RT-PCR for the detection, quantitation, and serotyping of dengue viruses from patient serum or plasma. We demonstrate that this diagnostic test is more analytically sensitive than a commonly used reference molecular assay, and is able to detect viral RNA and provide serotype information in specimens collected more than 5 days after fever onset and from patients who had already developed anti-DENV IgM antibodies. This unique combination of sensitivity and serotyping capability in a simple, single-reaction format represents a step forward in dengue diagnostics.

## Introduction

Dengue results from infection with one of four closely related serotypes of dengue virus (DENV), the most common vector-borne human pathogen worldwide. These serotypes are designated DENV-1, -2, -3, and -4, and they are transmitted by the mosquitoes *Aedes aegypti* and *Aedes albopictus*, which reside in tropical and sub-tropical areas of the world [1]. Infection with one serotype (primary infection) results in immunity to that serotype, but infection can occur with any of the remaining serotypes (secondary infection). Secondary DENV infection has been shown to be a significant risk factor for the development of severe disease, including dengue hemorrhagic fever (DHF) and dengue shock syndrome (DSS) [1]–[4]. Recent reports estimate that 230 million DENV infections occur annually, including 2 million cases of severe disease and 21,000 deaths [5]. Over 3.6 billion people live in endemic regions and are at risk for infection [5].

A large number of assays and testing platforms have been developed for the diagnosis of DENV infections, though available tests remain suboptimal [1]. Viral isolation continues to be performed in some laboratories, though the prolonged turn-around time precludes its utility for clinical diagnosis. Anti-DENV IgM antibodies do not become detectable until at least the third day of clinical illness, and IgM tests are not reliably positive until day five [6], [7]. The detection of IgM is not specific for acute dengue, as IgM may persist for two to three months following infection, and IgM against other flaviviruses can cross-react in some tests [8], [9]. IgG does not distinguish current from past infection, an important issue in endemic areas where secondary infection is common. Assays based on the detection of nonstructural protein 1 (NS1) tend to be specific for DENV infection, but the sensitivity of these assays ranges widely in published reports, from 24–93%, and varies based on the specific assay used and the infecting serotype [7], [10]. NS1 assays also show a roughly 20% decrease in sensitivity in the setting of secondary infection compared to primary infection [7],[11].

Reverse transcriptase-PCR (RT-PCR) for the detection of DENV has been in use since the 1990s, and many laboratories still use a hemi-nested RT-PCR that was originally developed in 1992 and modified by different groups over the intervening years [12], [13]. This assay requires two rounds of amplification followed by gel electrophoresis for amplicon detection; these steps result in an increased risk of contamination and also significantly limit the clinical utility of this assay, as the test requires one day or longer to perform. Numerous other RT-PCR assays have been reported in the literature and are used around the world, including real-time RT-PCR (rRT-PCR) and isothermal amplification techniques [14]–[25]. However, an international external quality control assessment, published in 2010, involving 37 laboratories performing 46 tests showed that 80% of these tests lacked sensitivity, specificity, or both [26].

The aim of this project was to develop a multiplex, rRT-PCR that allows for the detection, quantification, and serotype identification of DENV in a single reaction. For the purposes of this manuscript, the phrase “single-reaction” denotes the inclusion of all primers and probes in one reaction tube that accomplishes both reverse transcription and cDNA amplification. The single-reaction format was selected to improve workflow and decrease the opportunity for contamination. Single-reaction DENV RT-PCR assays utilizing real-time chemistry have been published previously, but to our knowledge only two have been directly compared to another nucleic acid amplification test, both to a version of the hemi-nested RT-PCR assay [12], [16]. Furthermore, in both of these reports, by Johnson, *et al.* and Chien, *et al.*, the single-reaction, multiplex assays proved less sensitive [12], [16], [27]. For this reason, the hemi-nested RT-PCR, previously modified to improve its sensitivity, was selected for use as reference [12].

This study describes the design and evaluation of a single-reaction, multiplex, quantitative rRT-PCR assay for the detection and serotyping of dengue viruses from patient samples. This assay targets the 5′ untranslated region (UTR) and capsid gene of DENV and utilizes four molecular beacons for detection and serotyping. Beacons were chosen for their high sensitivity and ability to differentiate sequences differing by as few as one or two bases [28]. This assay is shown to be more sensitive, both analytically and clinically, than the reference hemi-nested RT-PCR. It is also specific for dengue viruses, demonstrating no amplification when tested against the genomic RNA of other flaviviruses as well as samples from domestic (USA) patients without risk of DENV exposure. This assay represents an improvement over previously reported DENV rRT-PCRs as it maintains the sensitivity and serotyping capability of the reference molecular diagnostic test while also providing viral load monitoring and improved workflow in a simple, single-reaction format.

## Methods

### Dengue Virus Sequences and Assay Design

Publicly available DENV sequences were obtained from GenBank, accessed in August 2011. For DENV-1, -2, and -3, sequences deposited since 1990 were selected and up to 2 sequences per country per year were included (143 DENV-1 sequences, 181 DENV-2 sequences, 191 DENV-3 sequences). Due to the lower number of deposited sequences for DENV-4, all complete genome sequences available for this serotype (75 DENV-4 sequences) were included. Sequences listed as sylvatic strains were excluded. Segments of these genomes were aligned using MegAlign software (DNAStar; Madison, WI). A consensus sequence was generated for each serotype that showed bases conserved across ≥95% of sequences included in the alignment. This identified a region of the 5′ UTR and capsid gene that was highly conserved within each DENV serotype.

Using the 95% consensus sequence, Beacon Designer software (Premier Biosoft; Palo Alto, CA) was used to generate primer and probe sets directed against segments of the 5′ UTR and capsid gene for all four serotypes. These were tested using control genomic RNA for DENV-1, -2, -3, and -4, and based on initial results, primer and probe sets patterned on those for DENV-1 were generated for DENV-2, -3, and -4 using Primer3 software. The primer and probe sequences are listed in Table 1 and Table 2, respectively. The primers and probes were tested *in silico* using BLASTn to query the NCBI nucleotide database. Searches excluding the DENV group (taxid: 11052) were also performed to identify the best non-DENV sequence matches in the database as well as in the *Flaviviridae* family.

**Table 1:** Primer sequences for the DENV multiplex rRT-PCR.

Name	Primer Sequence (5′→3′)	Genomic Location
DENV-1, 2, 3 Forward	CAGATCTCTGATGAACAACCAACG	86–109
DENV-2 Forward C→T	CAGATCTCTGATGAATAACCAACG	87–110
DENV-3 Forward C→T	CAGATTTCTGATGAACAACCAACG	85–108
DENV-4 Forward	GATCTCTGGAAAAATGAAC	81–99
DENV-1, 3 Reverse	TTTGAGAATCTCTTCGCCAAC	DENV-1: 199-179, DENV-3: 198-178
DENV-2 Reverse	AGTTGACACGCGGTTTCTCT	171-152
DENV-2 Reverse A→G	AGTCGACACGCGGTTTCTCT	171-152
DENV-4 Reverse	AGAATCTCTTCACCAACC	190-173

Genomic locations are provided for the 5′ base of each primer based on reference virus sequences: DENV-1, US/Hawaii/1944 (GenBank: EU848545.1); DENV-2, New Guinea C Strain (GenBank: AF038403.1); DENV-3, strain H87 (GenBank: M93130.1); DENV-4, strain H241 (GenBank: AY947539.1). Predicted amplicon sizes are as follows: DENV-1, 114 bp; DENV-2, 85 bp; DENV-3, 114 bp; DENV-4, 110 bp.

**Table 2:** Probe sequences for the DENV multiplex rRT-PCR.

Channel	5′ Fluor	Probe Sequence (5′→3′)	3′ Quencher
Green	FAM	CGCGATCGCGTTTCAGCATATTGAAAGACGGATCGCG	BHQ-1
Yellow	CAL Fluor Orange 560	CGCGATCGCGTTTCAGCATATTGAAAGGCGGATCGCG	BHQ-1
Orange	CAL Fluor Red 610	CGCGATCCACGCGTTTCAGCATATTGATAGGATCGCG	BHQ-2
Red	Quasar 670	CGCGATCTTTCAGCATATTGAAAGGTGGTCGATCGCG	BHQ-2

Probes are listed by the channel in which signal is detected on the Rotor-Gene Q instrument. Underlined probe segments designate sequences complementary to the DENV consensus; segments on the 5′ and 3′ ends of the probe comprise the beacon stem.

### RT-PCR and Internal Control Assays

The DENV multiplex rRT-PCR was performed using the SuperScript III Platinum One-Step qRT-PCR kit (Invitrogen; Carlsbad, CA). Reaction mixtures were scaled from the manufacturer-recommended 50 µL volume to 25 µL per reaction. Each reaction contained 300 nM primers for DENV-1, -2, and -3 and 450 nM primers for DENV-4. Each probe was added to 600 nM in the final PCR reaction, and 5 µL of RNA eluate was added to each reaction. RT-PCR reactions were performed using the Rotor-Gene Q instrument (Qiagen; Valencia, CA). Cycling conditions were the following: 52°C for 15 min (RT step); 94°C for 2 min; 45 cycles of 94°C for 15 sec, 55°C for 20 sec, 60°C for 20 sec, and 68°C for 20 sec (run time, 136 min). Detection was performed in the green, yellow, orange, and red channels at 55°C; the gain was set at 10 for green, yellow, and orange, and at 5.33 for red. Four-step cycling was initially used to detect signal at different temperatures, but it was maintained as it showed improved sensitivity and curve generation compared to standard, three-step cycling (data not shown). During analysis, slope-correction was performed for each channel. Additionally, the first five cycles were cropped from the orange and red channel to improve baseline normalization. The threshold was set at 0.05 for green and yellow and 0.025 for orange and red. A positive result was considered any curve crossing this threshold prior to cycle 40. All results after cycle 40 were evaluated individually. The crossing threshold (Ct) value for each sample was recorded and the concentration of RNA calculated from a standard curve generated using quantified plasmid DNA (see below). Serotype was determined based on the pattern of signals obtained from the four DENV probes (Table 3).

**Table 3:** Probe signals for each DENV serotype.

		Channel			
		Green	Yellow	Orange	Red
**Serotype**	DENV-1	**+++**	**−**	**−**	**++**
	DENV-2	**+**	**++**	**−**	**−**
	DENV-3	**+**	**−**	**++**	**−**
	DENV-4	**−**	**−**	**−**	**++**

Any positive signal above the threshold is shown as (+). DENV-1 has the strongest and earliest signal in green of any serotype as (+++). Other positive signals are designated (++).

For the reference assay, a modified hemi-nested RT-PCR assay was used [12]. This assay was performed as previously described, except that reactions were scaled to 25 µL. All reactions were carried out in a DNA Engine Thermal Cycler (Bio-Rad; Hercules, CA). Briefly, One Step RT-PCR Kit (Qiagen; Valencia, CA) was used for the RT-PCR reactions. Twelve and a half pmoles of primers mD1 and D2 were added to the reaction, followed by 2.5 µL of RNA. RT-PCR conditions consisted of the following: an initial hold at 50°C for 30 min; one cycle at 95°C for 15 min, 55°C for 15 sec, and 72°C for 30 sec; 34 cycles of 95°C for 15 sec, 55°C for 15 sec, and 72°C for 30 sec; and a final 72°C extension for 10 min. For the nested PCR step, HotStarTaq DNA Polymerase (Qiagen; Valencia, CA) was used and the reaction mix was proportionally adjusted for a 25 µL final reaction volume. Twelve and a half pmoles of primers mD1, rTS1, mTS2, TS3, and TS4 were used per reaction, and 2.5 µL of RT-PCR reaction product was added. Cycling conditions were the following: 95°C for 15 min, then 25 cycles of 95°C for 15 sec, 55°C for 15 sec, and 72°C for 30 sec. Fifteen µL of the nested PCR reaction was loaded on 2.5% agarose gels and separated by electrophoresis. The gels were stained with ethidium bromide (0.4 µg/mL), bands visualized by UV light, and serotypes determined by product size (DENV-1 208 bp, DENV-2 119 bp, DENV-3 288 bp, DENV-4 260 bp).

A hydrolysis-probe-based rRT-PCR assay targeting the DENV NS5 gene (NS5 TaqMan) was adapted from a previously published report [12]. Reaction mixtures were scaled from 50 µL to 25 µL, and 5.0 µL of RNA was added to each reaction. All probes were labeled with FAM and utilized in a multiplex assay. RT-PCR was performed on the Rotor-Gene Q instrument, and cycling conditions were consistent with those described previously: 50°C for 30 min; 1 cycle of 95°C for 15 min, 50°C for 30 sec, and 72°C for 1 min; and 45 cycles of 95°C for 15 sec and 48°C for 3 minutes with continuous detection in the green channel.

A separate, internal control reaction for the detection of RNAse P was performed on the clinical samples from Nicaragua and Sri Lanka (see section entitled *Clinical Participants*). This assay has been previously described, though the protocol was modified for this study [29]. RT-PCR was carried out with SuperScript III Platinum One-Step qRT-PCR (Invitrogen; Carlsbad, CA) and the same cycling conditions as the multiplex DENV rRT-PCR were used. The reaction volume was scaled to 25 µL, and the final reaction mixture contained 200 nM forward and reverse primers and 100 nM probe.

### Reference Virus RNA

Genomic RNA from control strains of the four DENV serotypes, DENV-1 Hawaii 1944, DENV-2 New Guinea C strain, DENV-3 strain H87, and DENV-4 strain H241 were obtained from Vircell (Grenada, Spain). Genomic RNA of three strains of West Nile Virus (WNV, NY 1999; clinical isolate, previously reported as NAL strain; and B956), and a single strain each of Japanese Encephalitis Virus (JEV) and Tick-Borne Encephalitis Virus (TBEV) were obtained from the St. Orsola-Malphighi Hospital, Regional Reference Center for Microbiological Emergencies (Bologna, Italy) [30].

### Plasmid Generation, Quantitation, and Sequencing

The amplicons generated by the rRT-PCR assay for control strains of DENV and selected clinical specimens were cloned using the TOPO TA Cloning Kit with PCR 2.1 TOPO (Invitrogen; Carlsbad, CA). RT-PCR was performed as described above, except that reactions were carried out in a DNA Engine Thermal Cycler (Bio-Rad; Hercules, CA) without probes. Amplicons were detected by 2% agarose gel electrophoresis, and 2 µL of each PCR reaction was used in the cloning reaction. The presence of the cloned insert was confirmed by PCR using the Fermentas 2× PCR Master Mix (Fermentas; Glen Burnie, MD) and the same mix of DENV primers used in the rRT-PCR. Two µL of culture broth was boiled and cooled, then included in PCR reactions otherwise performed according to the manufacturer's recommendations. Cycling conditions included an initial hold at 94°C for 2 min, followed by 45 cycles of 94°C for 15 sec, 55°C for 30 sec, and 68°C for 30 sec. Amplicons were detected by 2% agarose gel electrophoresis. Plasmids were extracted using the GeneJET Plasmid Miniprep Kit (Fermentas; Glen Burnie, MD). The concentration of plasmid DNA was quantified using the AccuBlue High Sensitivity dsDNA Quantitation Kit (Biotium Inc.; Hayward, CA). Twenty-five-, 50-, and 100-fold dilutions were tested in triplicate. A standard curve was generated and the concentration of plasmid in the initial eluate calculated. Plasmids were sequenced by bi-directional dideoxynucleotide termination sequencing using M13 forward and reverse primers (Elim Biopharmaceuticals, Inc.; Hayward, CA).

### Multiplex rRT-PCR Linearity, Lower Limit of Detection, and Precision

The analytical evaluation of the DENV multiplex rRT-PCR was performed according to previously published recommendations [31]. For each serotype, linearity studies were performed on serial 10-fold dilutions of both quantified plasmid DNA and reference RNA. For the plasmid DNA, dilutions from 7.0 log_10_ copies/µL to 1 copy/µL were tested in triplicate on a single run. The concentrations of the reference RNA were originally quantified by the manufacturer in ng/µL of total RNA. Ten-fold dilutions from 1 ng/µL to 0.01 pg/µL RNA were tested in triplicate on a single run. Using the standard curve generated with dilutions of plasmid DNA, the concentration in DENV complementary DNA (cDNA) equivalents/µL was calculated for the highest concentration of RNA (1 ng/µL) for each serotype. Dilutions were performed on a single day for each serotype. The linear range was established by fitting a best-fit line to the data by regression analysis and included the range where the R^2^ value for this line was ≥0.99.

To establish the lower limit of 95% detection (95% LLOD), the lowest concentrations of RNA at which all five replicates were detectable during the linear range study were used as the starting point. Ten replicates of four, two-fold dilutions were tested in a single run. The 95% LLOD was then calculated using probit analysis. Analytical sensitivity for the multiplex assay was compared to the reference hemi-nested RT-PCR. The dilutions made for the test of linearity using RNA were added to the RT-PCR step of the hemi-nested PCR. Each 10-fold dilution was run in duplicate in the reference assay.

The precision of the multiplex rRT-PCR assay was determined using three dilutions of RNA controls (high positive, low positive, and limit of quantitation). These were performed as 5 replicates on 3 separate days. Fresh dilutions were made on the day of each run from aliquots of high concentration stocks (1 ng/µL, high positive). Intra- and inter-run variability was calculated from the log_10_-concentration of the samples, except where indicated.

### Multiplex rRT-PCR Specificity

Specificity was evaluated by testing genomic RNA from WNV, JEV, and TBEV isolates, as well as from the yellow fever 17D (YF-17D) vaccine strain. The JEV, TBEV, and WNV strains are described above. For YF-17D, genomic RNA (Vircell; Grenada, Spain) was tested at concentrations of at least 12,500 copies/µL and 250 copies/µL, as quantitated by the manufacturer. In addition, multiplex rRT-PCR assay specificity was determined by testing 80 patient plasma samples, 60 hepatitis C virus (HCV)-positive and 20 HCV-negative, sent to the Stanford Clinical Virology Laboratory for HCV viral load testing.

### Mixing Studies

Interference was evaluated by performing mixing studies using dilutions of quantitated plasmid. All dual-infection combinations were simulated using 2.0 log_10_ copies/µL of a given serotype mixed with serial, 10-fold dilutions of a second serotype extending from 5.0 to 2.0 log_10_ copies/µL. DENV-1 and -4 dual infections were differentiated from DENV-1 mono-infection based on Ct differences between the green and red channels.

### Clinical Participants

Seventy-four archived, de-identified samples from suspected dengue patients presenting within the first five days of clinical illness were tested. These samples were collected between September 9, 2009 and December 8, 2011 as part of the Nicaraguan Pediatric Dengue Cohort Study as well as a hospital-based study to assess risk factors for severe dengue in inpatients of the Infectious Diseases Ward of the Hospital Infantil Manuel de Jesús Rivera (Managua, Nicaragua). Study design and methods for both of these studies have been previously described [32]–[34]. All patients were clinically suspected of having dengue fever and had been tested using an earlier version of the hemi-nested RT-PCR assay [13]. RNA was extracted from separate aliquots of plasma stored at −80°C and tested, during a single freeze-thaw cycle, using the DENV multiplex rRT-PCR and the reference assay.

Forty de-identified plasma samples from children presenting to the Lady Ridgeway Hospital (Colombo, Sri Lanka) with an acute febrile illness were also tested. These samples were collected prospectively from March 18 to May 28, 2012. These patients were clinically diagnosed with DF, DHF, or DSS according to the WHO 1997 recommendations [4]. Patients were tested with the Hexagon GmbH Dengue assay (HUMAN Diagnostics, Wiesbaden, Germany), which is a rapid assay detecting anti-DENV IgM and IgG antibodies. Patients were diagnosed with primary dengue if they were IgM positive and IgG negative. Patients with only detectable IgG were judged to have secondary dengue infection. Patient data sent with the sample included patient age, clinical diagnosis (DF, DHF, or DSS), and whether the patient died as a result of this infection.

### Nucleic Acid Extraction of Clinical Specimens

Nucleic acid extraction was performed using the QIAamp Viral RNA Mini Kit (Qiagen; Valencia, CA). All extractions were carried out according to the manufacturer's recommendations. Extractions were performed using 140 µL of patient plasma, eluted into 60 µL of buffer AVE. The quantitation of clinical specimens in cDNA equivalents/mL serum or plasma took into account these extraction and elution volumes and was calculated using the following formula: (cDNA equivalents/µL * 60 µL)/(0.14 mL).

### Ethics

The protocols for the Nicaraguan Pediatric Dengue Cohort Study and Pediatric Hospital-based Dengue Study were reviewed and approved by the Institutional Review Boards (IRB) of the University of California, Berkeley, and the Nicaraguan Ministry of Health. Parents or legal guardians of all subjects provided written informed consent, and subjects 6 years of age and older provided assent. The IRB at Stanford University waived review of this study, as samples were pre-collected and de-identified.

### Statistics

Basic statistical analysis including the calculation of means and standard deviation was performed using Excel software (Microsoft; Bellevue, WA). Two-tailed Fisher's exact tests, t-tests, and kappa calculations were performed using GraphPad software (GraphPad; La Jolla, CA). Probit analysis was performed using SPSS (IBM; Armonk, NY).

## Results

### Linearity and Lower Limit of Detection

**Figure 1:**
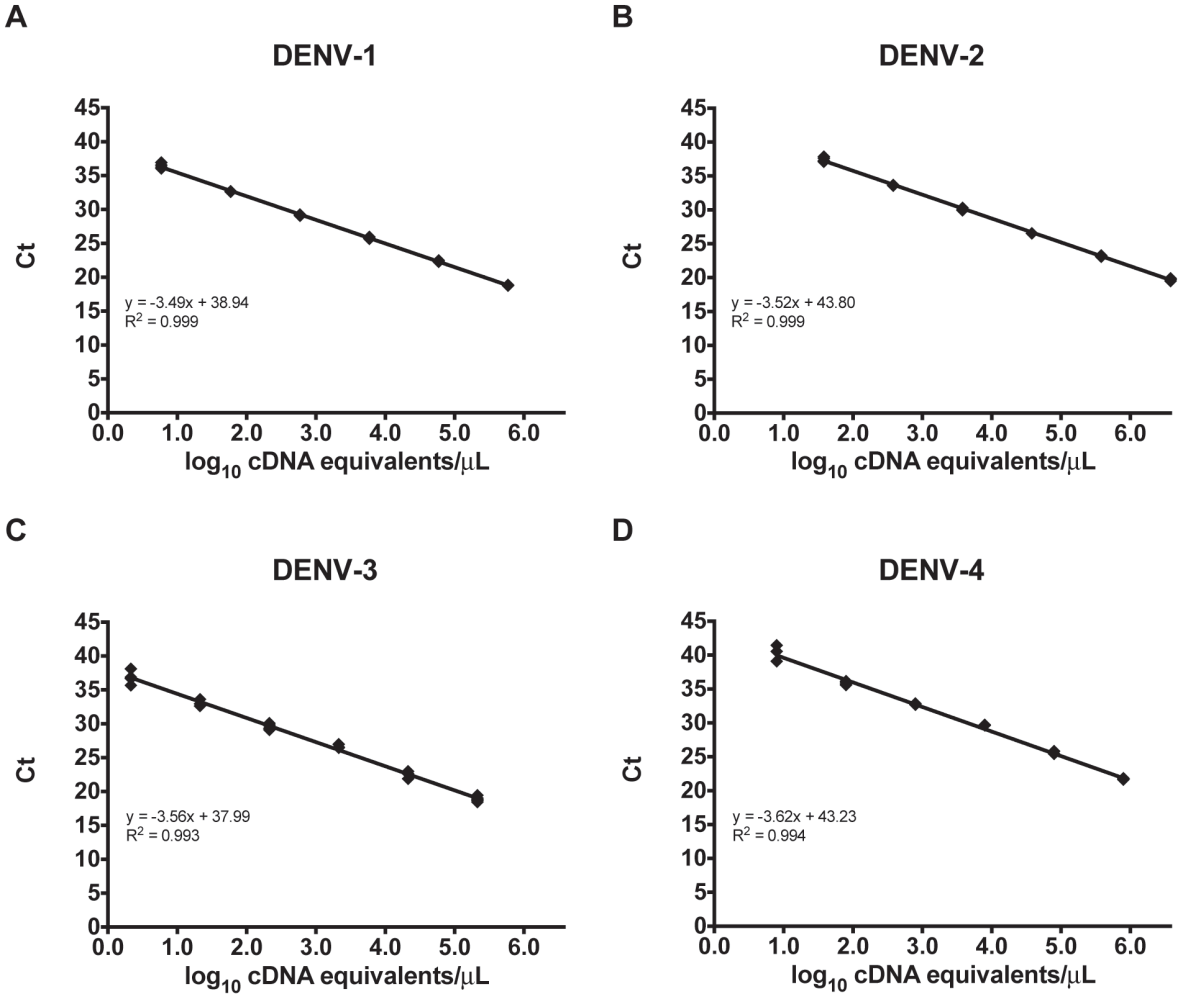
The linear range for the multiplex rRT-PCR assay is shown for DENV-1 (A), DENV-2 (B), DENV-3 (C), and DENV-4 (D).

The primers and probe sets used in the multiplex assay are shown in Tables 1 and 2, respectively. The probes differ by one to four bases between the different serotypes and are listed by the channel in which they are detected. The pattern of signal obtained from the different probes for each serotype is displayed in Table 3. Using DENV reference RNA dilutions, the linear range for each serotype extended from 1.0 ng RNA/µL to 0.01 pg RNA/µL. Based on standard curves generated with dilutions of plasmid DNA, the concentration in DENV cDNA equivalents/µL for the highest concentration of RNA was determined for each serotype. The linear range of the multiplex assay therefore corresponds to the following cDNA equivalents/µL: DENV-1, 5.8 to 0.8 log_10_; DENV-2, 6.6 to 1.6 log_10_; DENV-3, 5.3 to 0.3 log_10_; DENV-4, 5.9 to 0.9 log_10_ (Figure 1). Using plasmid DNA, the linear range for each serotype extended from 7.0 to 1.0 log_10_ copies/µL for DENV-1, -2, and -3, and from 7.0 to 2.0 log_10_ copies/µL for DENV-4 (data not shown).

The lower limit of 95% detection (LLOD) was determined for each serotype in the multiplex assay by probit analysis using reference RNA dilutions. The LLOD was calculated to be 0.3 cDNA equivalents/µL for DENV-1, 13.8 for DENV-2, 0.8 for DENV-3, and 12.4 for DENV-4. For comparison, duplicates of the same RNA dilutions were run, on the same day, using the hemi-nested RT-PCR reference assay. The lowest concentrations detected by the reference assay were 6 cDNA equivalents/µL (2 of 2 replicates) for DENV-1, 3800 (2 of 2 replicates) for DENV-2, 21 (1 of 2 replicates) for DENV-3, and 79 (1 of 2 replicates) for DENV-4. The multiplex rRT-PCR assay, therefore, was more analytically sensitive for each serotype than the reference assay.

We initially intended to compare our multiplex rRT-PCR with a multiplex, hydrolysis probe-based rRT-PCR targeting the DENV NS5 gene (NS5 TaqMan). Therefore, this NS5 TaqMan assay was also evaluated using duplicates of 10-fold RNA dilutions. The lowest concentrations detected by the NS5 TaqMan assay were 76,000 cDNA equivalents/µL (2 of 2 replicates) for DENV-1, 89,000 (2 of 2 replicates) for DENV-2, 224 (1 of 2 replicates) for DENV-3, and 16 (1 of 2 replicates) for DENV-4. Given the comparatively poor analytical sensitivity of the NS5 TaqMan assay, it was not tested in further experiments.

### Precision

Both intra- and inter-run precision were calculated for each serotype at three concentrations (high-positive, low-positive, and limit-of-quantitation) using reference RNA. These calculations were performed using the log_10_ cDNA equivalents/µL for each sample, except for DENV-3 at the limit of quantitation, where the mean concentration and precision calculations were expressed on the linear scale. Each mean concentration as well as intra- and inter-run precision are shown in Table 4.

**Table 4:** Precision analysis of the DENV multiplex rRT-PCR.

		Inter-Run Variability			Intra-Run Variability		
		Mean	SD	%CoV	Mean	SD	%CoV
		log_10_ cDNA equivalents/µL			Range. log_10_ cDNA equivalents/µL	Range	Range
**DENV-1**	High-positive	5.77	0.03	0.6	5.75–5.79	0.01–0.04	0.3–0.7
	Low-positive	2.67	0.03	1.3	2.65–2.69	0.02–0.04	0.7–1.7
	Limit of quantitation	0.49	0.12	23.7	0.4–0.54	0.07–0.12	17.6–22.0
**DENV-2**	High-positive	6.60	0.06	0.9	6.57–6.67	0.02–0.05	0.3–0.8
	Low-positive	3.57	0.06	1.7	3.51–3.60	0.03–0.07	0.7–1.8
	Limit of quantitation	1.47	0.11	7.8	1.41–1.59	0.04–0.09	3.0–6.5
**DENV-3**	High-positive	5.18	0.18	3.4	5.05–5.33	0.11–0.18	2.1–3.5
	Low-positive	1.99	0.14	7.1	1.85–2.10	0.09–0.12	4.3–5.7
	Limit of quantitation*	0.73	0.45	61.8	0.56–0.99	0.35–0.56	56.9–61.3
**DENV-4**	High-positive	5.85	0.06	1.0	5.78–5.90	0.03–0.05	0.5–0.8
	Low-positive	2.88	0.06	2.0	2.85–2.93	0.02–0.07	0.7–2.4
	Limit of quantitation	1.99	0.10	5.2	1.94–2.04	0.06–0.15	3.1–7.5

* Expressed as cDNA equivalents/µL.

SD, standard deviation; %CoV, percent coefficient of variation.

### Mixed Infection

DENV plasmids were mixed at known concentrations to simulate infections involving two different serotypes. Dual infections were detected for all serotype combinations when mixed at equal concentrations (data not shown). For each serotype, tested at the limit of quantitation, amplification was inhibited by 10 to 1000-fold higher concentrations of the competing serotype.

### Specificity

Genomic RNA from one strain each of JEV and TBEV, three strains of WNV, and high and low concentrations of YF-17D showed no detectable signal in the multiplex rRT-PCR. Eighty archived patient samples that had previously been sent for HCV testing (60 positive and 20 negative samples) were also tested. The positive samples had a median HCV viral load of 6.05 log_10_ copies/mL (range 1.63–7.66 log_10_ copies/mL). These samples also showed no detectable signal in the multiplex rRT-PCR assay.

As a further test of specificity, the primers and probes were tested *in silico* by querying the NCBI nucleotide database for related sequences. The best, non-DENV matches for the primer sequences showed 75–100% coverage but included a diverse group of organisms unlikely to be identified in dengue patients (e.g. *Schizophyllum commune*, 100% coverage for the DENV-4 reverse primer). The best primer matches among members of the *Flaviviridae* showed only 50–68% coverage. No organism represented the best match for both the forward and reverse primers for any of our primer pairs. For the probes, certain JEV strains had 56–91% coverage, though these sequences would not be predicted to amplify using the primers in this assay.

### Clinical Samples

Seventy-four archived plasma samples that were collected from Nicaraguan children within the first five days of a dengue-like illness were tested using both the multiplex rRT-PCR and the reference hemi-nested assay. The mean day of illness for patients in this group was 3.39 (standard deviation, 1.25). The two assays showed good agreement (Table 5, kappa = 0.737). The multiplex rRT-PCR was positive in 100% (69/69) of samples with detectable DENV RNA in the reference assay. There was complete agreement in the serotyping calls between the two assays (22 DENV-1, 12 DENV-2, 35 DENV-3). An additional two samples had detectable DENV RNA by the multiplex rRT-PCR but were negative by the reference assay (1 DENV-1, 1 DENV-3). Three samples tested negative by both assays.

**Table 5:** Comparison of samples collected from patients in Nicaragua presenting within the first five days of a dengue-like illness.

DENV	Hemi-Nested RT-PCR		
Multiplex			
rRT-PCR	Positive	Negative	Total
Positive	69	2	71
Negative	0	3	3
Total	69	5	74

Forty plasma samples were obtained from children in Sri Lanka with a clinical diagnosis of dengue, collected prospectively during a single dengue season. Thirty-six patients had detectable anti-DENV IgM antibodies at diagnosis (90%). These samples were tested using both the multiplex rRT-PCR and the reference hemi-nested assay. The multiplex rRT-PCR was positive in 95.0% of patients (38/40, 37 DENV-1 and 1 DENV-3), whereas the reference assay was positive in only 40.0% (16/40, 16 DENV-1). All samples detected by the reference assay were also detected by the multiplex rRT-PCR. In the 36 patients with detectable IgM antibodies, the multiplex rRT-PCR was positive in 97.2% (35/36) whereas the reference assay was positive in only 44.4% (16/36). Amplicons from four multiplex rRT-PCR-positive, reference assay-negative samples were cloned and sequenced, confirming the specific amplification of DENV RNA. The samples contained similar levels of RNAse P RNA, verifying adequate extraction and the absence of RT-PCR inhibitors (data not shown).

The composition of the groups of patients from Sri Lanka who were positive by the DENV multiplex rRT-PCR alone and positive by both assays is shown in Table 6. These two groups were generally similar. However, patients who tested positive only by the multiplex rRT-PCR were more likely to have secondary dengue (p = 0.047), and they had a lower average viral load (p = 0.0002). Two of the forty patients tested negative by both assays. One patient was clinically diagnosed with DF and had a positive IgM test on day 5 of illness. The second patient was diagnosed with an undifferentiated febrile illness and tested IgM negative on day seven of illness. A single patient died; their viral load was 3.2 log_10_ cDNA equivalents/mL on day of illness seven.

**Table 6:** Comparison of patients from Sri Lanka that tested positive only by the multiplex rRT-PCR and those that tested positive by both the multiplex rRT-PCR and reference assays.

	Multiplex rRT-PCR Positive	Multiplex rRT-PCR Positive	
	Reference Method Negative	Reference Method Positive	p-value
Patients, n (%)	22 (55)	16 (40)	ND
Age, mean (SD)	7.1 (3.6)	6.1 (4.0)	0.43
Day of Illness, mean (SD)	5.8 (1.6)	6.1 (1.2)	0.53
IgM Positive, n (%)	19 (86)	16 (100)	0.25
Primary Dengue, n (%)	7 (32)	11 (69)	0.047
Illness Severity, n (%)			
DF	15 (68)	6 (38)	0.10
DHF/DSS	7 (32)	10 (62)	
Any ICU Care, n (%)	4 (18)	7 (44)	0.15
Viral Load, mean (SD)*	2.98 (0.61)	4.4 (1.41)	0.0002

* Viral Load expressed as log_10_ cDNA equivalents/mL of patient plasma.

ND, not determined; SD, standard deviation.

Overall, the multiplex rRT-PCR detected 100% (85/85) of the samples detected by the reference assay, with perfect serotype agreement. Furthermore, the multiplex rRT-PCR identified DENV RNA in 24 additional clinical specimens from patients with known recent DENV infection.

## Discussion

This study describes the development of a single-reaction, multiplex rRT-PCR for the detection, quantitation, and serotyping of dengue viruses from patient samples. Analytical and clinical evaluation of the assay was performed using reference RNA that included the four DENV serotypes and other closely related flaviviruses, as well as clinical specimens from Nicaragua, Sri Lanka, and the United States.

The DENV multiplex rRT-PCR was compared to a version of a hemi-nested RT-PCR that has been in use for at least two decades and continues to be performed in laboratories throughout the world [12], [13], [25]. To our knowledge only two single-reaction rRT-PCR assays with serotyping capability have been directly compared to another nucleic acid amplification test, and in both reports, the hemi-nested RT-PCR was used as reference. In both cases, the hemi-nested assay proved more sensitive [12], [16]. Johnson, *et al.* details the evaluation of a hydrolysis probe-based multiplex assay targeting different regions of the DENV genome for each serotype. The analytical sensitivity of the assay was 10-fold lower than the reference assay, possibly resulting from the use of a relatively low Ct cutoff (<36 cycles) due to background cross-reactivity between primers and probes in no-template control reactions [16]. In the second report, Chien, *et al.* describes the development of another hydrolysis probe-based multiplex targeting the 3′ region of the NS5 gene using primers previously developed for flavivirus sequencing [12]. This NS5 TaqMan assay demonstrated 91% clinical sensitivity compared to the reference assay when cultured isolates and high-titer acute phase serum specimens were tested. The NS5 TaqMan assay has been used as a comparator in other reports and serves as one component of a larger multiplex assay to detect eight different flaviviruses [14], [22], [35]. We therefore initially selected this assay to compare to our multiplex rRT-PCR. In our hands, this assay was at least 200-fold less analytically sensitive than the hemi-nested RT-PCR using reference RNA for DENV-1 and -3. Because of this relatively poor sensitivity, we did not utilize the NS5 TaqMan assay as reference.

In this study, we demonstrate that the DENV multiplex rRT-PCR is more analytically sensitive than the reference hemi-nested RT-PCR. Consistent with this finding, DENV RNA was detectable in more patient specimens using the multiplex rRT-PCR (95.6%; 109/114) than the reference assay (74.6%; 85/114). This difference is primarily accounted for by viral RNA detection in specimens positive for DENV IgM (22 specimens). In previous studies, such samples are often not tested, given low rates of RT-PCR positivity, and assays are typically evaluated using samples positive by viral isolation [12], . In the report by Kong, *et al.*, IgM-positive samples were tested, but only 69% were positive for DENV RNA by their assay. The rate of detection dropped to below 10% by day five of illness [17]. In contrast, our DENV multiplex rRT-PCR maintained a sensitivity of 97.2% (35/36) for the diagnosis of dengue even after the development of IgM antibodies, which may lengthen the period of time during which a patient can receive a virologically confirmed, serotype-specific diagnosis of dengue. Despite this sensitivity, the DENV multiplex rRT-PCR was specific for dengue viruses and did not amplify related flaviviruses, even at very high viral loads.

A number of other multiplex, serotype-specific rRT-PCR assays have previously been described in the literature, but they all have specific limitations compared to the assay reported here. An RT-PCR-ligase detection reaction for DENV compared favorably to the NS5 TaqMan rRT-PCR, but this assay involves a complicated design including lab-developed microarray detection that is not commonly used even in very sophisticated molecular virology laboratories [14]. The hydrolysis probe-based rRT-PCR assays reported by Kong, *et al.* and Lai, *et al.* showed promising results, but were designed using very small numbers of DENV sequences, raising concerns regarding their generalizability [17], [18]. Indeed, the assay by Lai, *et al.* was used in a study of DENV and chikungunya virus in Tanzania, where none of the 71 patients with presumptive, acute DENV infections based on IgM detection had detectable DENV RNA. The median day of illness for these patients was 4.5 (IQR 3–14) [36]. Multiplex rRT-PCR assays that utilize melting curves for serotyping do not clearly distinguish between serotypes, whereas other assays require multiple reactions to achieve accurate serotyping [15], [19], [23]. Recently, the United States Food and Drug Administration (FDA) approved a single-reaction, multiplex rRT-PCR developed by the Centers for Disease Control and Prevention (CDC), but this assay was not available for evaluation at the time of our study [37].

Further benefits of the DENV multiplex rRT-PCR include its single-reaction set-up and a reaction set-up-to-result time that is much shorter than the hemi-nested PCR (3 hrs. vs. 10 hrs.). The real-time design of this assay allows for faster results than the reference assay, though it does not provide a result as rapidly as point-of-care diagnostics such as NS1 and IgM lateral flow detection kits. These rapid antigen and antibody assays do not require the laboratory infrastructure necessary to perform RT-PCR, but they also cannot provide the amount of information available from a serotype-specific multiplex rRT-PCR [9]. Assay cost has also been cited as another advantage of rapid diagnostics. As reported here, the cost of the DENV multiplex rRT-PCR is 2.60 USD per reaction plus 4.00 USD for RNA extraction, which compares favorably to the cost of the rapid assay utilized in this study (6.00 USD, Dr. Abeynayake, personal communication).

The assay described herein was designed as a quantitative test for DENV. Though the clinical utility of viral load testing in the management of patients with DF remains unclear, previous studies have shown that patients with higher viral loads are at an increased risk for severe disease [38], [39]. Vaughn, *et al.* showed that peak viremia, when identified, correlated with severe disease in children infected with DENV-1 and DENV-2 [38]. It has also been shown that detectable viremia on the day of defervescence identifies patients at an increased risk for DHF [40]. In addition, studies have shown higher levels of peak viremia in secondary infection but also suggest that there may be differences based on the serotype of the causative DENV strain [41], [42]. Identifying clinically meaningful thresholds by quantitative rRT-PCR, may, then, be serotype-specific.

Mixed infections with two or more DENV serotypes have been documented in the literature since 1985, the first of which involved a case from the 1982 DENV outbreak in Puerto Rico [43]. Mixed infections have since been reported in other regions that experience the co-circulation of multiple DENV serotypes, and the rates of such cases have varied from 0 to 56.8% of patients presenting with symptomatic DF [44]–[49]. All combinations of dual infections with two DENV serotype were simulated in this study by mixing known concentrations of plasmid cDNA. The DENV multiplex rRT-PCR was able to detect both serotypes in all combinations when mixed at equal concentrations. The clinical significance of mixed infections remains unclear, though it does not appear that patients with such infections are at increased risk of developing severe dengue [43], [47].

This study involved a rigorous laboratory evaluation of the DENV multiplex rRT-PCR, which was designed using more than 500 DENV sequences from varied geographic locations over the past twenty years. While this design strategy should improve the generalizability of these results, the study is limited by the absence of clinical samples positive for DENV-4. As with any nucleic acid-based detection method, this assay may also be affected by the emergence of diversity in the target sequences. Though the assay is quantitative, further studies using serial samples collected over the course of a patient's illness will need to be performed in order to show that it can be used for the monitoring of dengue viral loads.

In summary, this study describes a single-reaction, multiplex rRT-PCR for the detection, quantitation, and serotyping of dengue viruses from patient serum or plasma. This assay is more analytically sensitive than the reference molecular assay, and clinically, was able to detect and serotype virus late in the clinical course of patients with detectable anti-dengue IgM antibodies. This assay makes an important contribution to available dengue diagnostics and warrants further study in additional cohorts of dengue patients.

## Supporting Information

Checklist S1 STAndards for the Reporting of Diagnostic accuracy studies (STARD) checklist.
(DOC)

Figure S1 STAndards for the Reporting of Diagnostic accuracy studies (STARD) flowchart.
(EPS)
